# Short-Term Outcomes and Quality of Life Following Minimally Invasive Esophagectomy in a Tertiary Care Center in Southern India

**DOI:** 10.7759/cureus.49245

**Published:** 2023-11-22

**Authors:** Vivek G Nath, Senthilkumaran Govindaraj Raman, Thiruvarul M, Kalyanasundarabharathi V C, Mathews Micheal, Joel Kumar Earjala, Lokeshwaran A, Arun Raja A, Aravindan U

**Affiliations:** 1 Department of Surgical Gastroenterology & GI Oncology, Thanjavur Medical College, Thanjavur, IND

**Keywords:** adenocarcinoma esophagus, minimal invasive gastrointestinal surgery, esophageal squamous cell carcinoma (scc), dysphagia', heath related quality of life, video assisted thoracoscopic surgery, carcinoma esophagus

## Abstract

Introduction: With the advent of multimodality therapy and minimally invasive surgical approaches, patients suffering from carcinoma esophagus are showing promising outcomes. Hence, the frontier needs to be widened to assess the postoperative quality of life (QoL) of those surviving carcinoma esophagus. The objective of the study was to determine the short-term outcomes of minimally invasive esophagectomy (MIE) /hybrid esophagectomies in carcinoma esophagus and the organ-specific QoL in survivors of MIE for carcinoma esophagus, and to compare health-related QoL in patients following MIE for carcinoma esophagus with the general population.

Methods and materials: A longitudinal study design was used to evaluate the short-term postoperative outcomes of patients undergoing MIE for carcinoma esophagus between July 1, 2021, to July 15, 2022, and analyze the QoL of those patients who survived at one year without tumor recurrence. QoL was assessed using the European Organization for the Research and Treatment of Cancer (EORTC) QoL Questionnaire (EORTC QLQ-C30) and the EORTC QoL Questionnaire - Oesophageal Cancer Module (EORTC QLQ-OES18).

Results: A total of 15 patients who underwent minimal invasive/hybrid esophagectomy for esophageal carcinomawere included. Of these, 13 patients underwent hybrid esophagectomy while two patients underwent thoraco-laparoscopic esophagectomy. Squamous cell carcinoma was observed as the most common histological variant (60%) while 33% were adenocarcinoma and 6.7% lymphoma. The most common site of the tumor was the lower one-third esophagus (60%). Nine out of 15 patients developed postoperative complications needing prolonged ICU stay. One major anastomotic leak as well as one conduit necrosis was observed among 15 cases operated. Median length of hospital stay was 16 (IQR 12-24). QoL was assessed among 12 patients at the one-year follow-up excluding mortality cases and patients with tumor recurrence. The patients following MIE for carcinoma esophagus were observed to have low scores in physical functioning, role functioning, and social function when compared with the general population. Cognitive functioning and emotional function were not found to be significantly different. No statistically significant difference was observed in the global health status among the two groups. There was no significant difference found in the general symptoms score comparison of the MIE patients with the general population. When it comes to organ-specific symptom scales, reflux was observed as a major issue among the patients who survived carcinoma esophagus after undergoing MIE. Dysphagia and dry mouth received low scores suggestive of minor issues. Though analysis of global health QoL scores of those with postoperative complications and those who had uneventful recovery at one year revealed a higher score for the latter, it was not statistically significant.

Conclusion: Postoperative complications can prolong hospital stay, delay resuming normal work, and affect the global QoL of patients compared with those who recovered uneventfully. Physical and role functions were observed to be deficient among survived patients when compared with the normal population. Nutritional prehabilitation, cutting-edge surgical practice including minimally invasive techniques, minimizing deviation from normal postoperative recovery by high-quality ICU care, and postoperative rehabilitation are the cornerstones to ensure better QoL.

## Introduction

Esophageal cancer is the eighth most common cancer worldwide according to the World Cancer Research Fund International. It is the seventh most common cancer in men and the 13th most common cancer in women [1]. According to the Surveillance, Epidemiology, and End Results (SEER) database, the estimated number of new carcinoma esophagus cases in 2023 is 21,560 with an estimated cancer-related death of 16,120 in the United States. In 2020, there were an estimated 50,379 people living with esophageal cancer in the United States. Five-year relative survival (2013-2019) for those affected with carcinoma esophagus is 21.7%, with five-year relative survival for localized cancer being 48.8% [2].

In the Indian population, as per WHO, in 2018, esophageal cancer was the sixth most common cancer with an incidence of 5.04%. It is the fifth most common cancer in males and the sixth most common cancer in females. The male-to-female ratio in India is 2.4:1 [3]. Unlike Western data, in the Indian subcontinent, squamous cell carcinoma (SCC) continues to be the leading histological type of esophageal carcinoma. Adenocarcinoma is more confined to the lower esophagus and gastroesophageal junction due to its etiological connection with Barret’s esophagus. SCC can arise from any part of the esophagus, as the entire length of the esophagus is covered with squamous epithelium [4].

Multimodality treatment has become the standard of care for esophageal cancer in recent times. Promising results from various trials emphasize neoadjuvant chemoradiation [5], followed by salvage esophagectomy in locally advanced cases, which in fact has changed the survival from dismal figures to an encouraging 21% [6].

Minimally invasive radical esophagectomy with two-field lymphadenectomy adds to this improved survival with fewer postoperative complications, especially respiratory morbidity [7]. As the relative survival is showing improvement, the frontier must be widened in assessing the quality of life (QoL) of the survivors. It is worth noting that short-term surgical outcomes are complimentary with the QoL of the survivors.

In this study, we aim to determine the short-term morbidity and QoL of carcinoma esophagus cases managed with minimally invasive esophagectomy (MIE). The objective of this study is to: (i) determine the short-term postoperative outcomes in minimally invasive/hybrid esophagectomies in carcinoma esophagus; (ii) determine the organ-specific quality of life in survivors of MIE for carcinoma esophagus at one year; (iii) To compare health-related QoL in patients surviving at one year following MIE for carcinoma esophagus with general population. 

## Materials and methods

Study design

A longitudinal study design was used to evaluate the short-term postoperative outcomes of patients undergoing MIE for carcinoma esophagus between July 1, 2021, and July 15, 2022, and analyzed the QoL of those patients who survived at one year without tumor recurrence. QoL was assessed using the European Organization for the Research and Treatment of Cancer (EORTC) QoL Questionnaire (EORTC QLQ-C30) and the EORTC QoL Questionnaire - Oesophageal Cancer Module (EORTC QLQ-OES18). The study was approved by the Institutional Ethical Committee of Thanjavur Medical College (Study no:152(f)/2021/IEC, approval no: 674/2021). Informed written consent was obtained from all participants.

The study included all cases of MIE for carcinoma esophagus between July 1, 2021, and July 15, 2022, in the Surgical Gastroenterology Department, Thanjavur Medical College, Tamil Nadu, India. The study population includes all diagnosed cases of carcinoma esophagus localized to the middle or lower esophagus, or junctional (Siewert type 1), undergoing MIE/hybrid esophagectomy. Diagnosis of carcinoma esophagus was arrived by endoscopy with biopsy and staging was done with contrast-enhanced CT of the thorax and abdomen with pelvis with or without positron emission tomography (PET) scan as indicated. Those patients having dysphagia grade four or above received either nasogastric tube insertion or a feeding jejunostomy to ensure nutrition. Patients received neoadjuvant chemoradiotherapy as indicated after discussion in the multidisciplinary forum, based on the stage of the disease adhering to the National Comprehensive Cancer Network® (NCCN®) guidelines. Post-neoadjuvant therapy, response evaluation, and restaging were done in eight weeks. Patients were offered either hybrid esophagectomy (video-assisted thoracoscopic (VAT) esophagectomy plus laparotomy or Thoraco-laparoscopic esophagectomy as per institutional protocol. 

Study tools

Patient’s demographic characteristics, clinical profile, intra- and perioperative parameters, postoperative recovery trends, postoperative in-hospital complication details, reintervention details, etc. were carefully recorded. Patients with poor long-term outcomes like cancer recurrence were excluded from the assessment of QoL. QoL was assessed at 12 months postoperatively, in view of giving an adequate time gap for recovery from major surgery and chemoradiation. We used EORTC-QOL-C30 and EORTC QLQ-OES18 for QoL assessment.

EORTC-QOL-C30 (Version-3) Tamil Version

QoL was assessed using a validated questionnaire. The QLQ-C30 is composed of both multi-item scales and single-item measures. These include five functional scales, three symptom scales, a global health status/QoL scale, and six single items. Each of the multi-item scales includes a different set of items; no item occurs in more than one scale. All the scales and single-item measures range in score from 0 to 100. A high scale score represents a higher response level. Thus, a high score for a functional scale represents a high/healthy level of functioning, a high score for the global health status/QoL represents a high QoL, but a high score for a symptom scale/item represents a high level of symptomatology/problems [8].

EORTC QLQ-OES18, Tamil Translated and Validated

EORTC QLQ-OES18, was a supplementary questionnaire module to be employed in conjunction with the QLQ-C30. The QLQ-OES18 incorporates four multi-item scales to assess dysphagia, eating, reflux, and pain. In addition, six single items assess trouble swallowing saliva, choking when swallowing, dry mouth, trouble with taste, trouble with coughing, and trouble talking [9]. A high score on a symptom scale represents a high level of symptoms.

Statistical analysis

Data analysis was performed using IBM SPSS Statistics for Windows, Version 20.0 (Released 2011; IBM Corp., Armonk, New York, United States). Quantitative variables with a normal distribution were expressed as mean (SD) and those with a non‐parametric distribution as median (interquartile range (IQR)) values. Categorical data were expressed as frequencies and percentages. The χ2 test was used to compare categorical data and proportions, and Student's t test or the Mann-Whitney U test, as appropriate, to compare continuous variables.

## Results

Between July 1, 2021, and July 15, 2022, a total of 15 patients underwent minimal invasive/hybrid esophagectomy for esophageal carcinoma in our department. Among them, 10 (66.6%) were males and five (33.3%) were females. An overview of demographic data and baseline characteristics is shown in Table 1.

**Table 1:** Demographic data and baseline characteristics ASA, American Society of Anesthesiologists; BMI, body mass index; IQR, interquartile range; ECOG, Eastern Cooperative Oncology Group; SCC, squamous cell carcinoma; OGJ, esophagogastric junction; COPD, chronic obstructive pulmonary disease; RT, radiotherapy. Quantitative variables with a normal distribution were expressed as mean± SD and those with a non‐parametric distribution as median (IQR) values. Categorical data were expressed as frequencies (n) and percentages (%).

Characteristics	Patients (N=15)
Sex, n (%)	
Male	10 (66.7)
Female	5 (33.3)
Age (years), mean± SD	50.4 ± 13.0
BMI (kg/m^2^), median (IQR)	19 (17-25)
ASA, n (%)	
1	4 (26.7)
2	11 (73.3)
3	0 (0)
ECOG score, n (%)	
0	3 (20)
1	12 (80)
2	0
3	0
Histopathological examination, n (%)	
SCC	9 (60)
Adenocarcinoma	5 (33.3)
Lymphoma	1 (6.7)
Site of tumor, n (%)	
OGJ, Siewert 1	2 (13.3)
Lower 1/3^rd^	9 (60)
Lower + OGJ	3 (20)
Middle 1/3^rd^	1 (6.7)
Diabetes mellitus, n (%)	
Yes	1 (6.7)
No	14 (93.3)
Hypertension, n (%)	
Yes	3 (20)
No	12 (80)
Bronchial asthma, n (%)	
Yes	1 (6.7)
No	14 (93.3)
COPD, n (%)	
Yes	3 (20)
No	12 (80)
Smoking, n (%)	
Yes	9 (60%)
No	6 (40%)
Prior RT, n (%)	
Yes	14 (93.3)
No	1 (6.7)

Details of surgical procedures, perioperative parameters, postoperative recovery, and major short-term (in-hospital) outcomes of minimally invasive esophagectomy have been enlisted in Table 2. Out of 15 patients, nine (60%) developed major postoperative complications including pulmonary morbidity. One patient, who received a colonic conduit, developed conduit necrosis and later progressed to multiple organ dysfunction syndrome (MODS) and death. Seven (46.7%) patients needed continuous positive airway pressure (CPAP) ventilation in the postoperative period, out of which two required reintubation and mechanical ventilation. Persistent hyponatremia was observed in one patient, who needed steroids and vasopressin receptor antagonists.

**Table 2:** Details of procedures, perioperative parameters, postoperative recovery, complications post MIE for carcinoma esophagus LN, lymph node; IQR, interquartile range; AKI, acute kidney injury; AF, atrial fibrillation; MODS, multi-organ dysfunction; CPAP, continuous positive airway pressure, major anastomotic leak, grade 3 or above [10]. Quantitative variables with a normal distribution were expressed as mean± SD and those with a non‐parametric distribution as median (IQR) values. Categorical data were expressed as frequencies (n) and percentages (%).

Medical details	Patients (N=15)
Procedure	
Thoraco-laparoscopic esophagectomy, n (%)	2 (13.3)
Hybrid esophagectomy, n (%)	13 (86.7)
Conduit used	
Stomach, n (%)	14 (93.3)
Colon, n (%)	1 (6.7)
Duration of surgery (minutes), mean ± SD	344 ± 83.2
Median blood transfusion (units), median (range)	1 (0-3)
Median blood loss (ml), median (IQR)	200 (150-220)
LN harvested, mean± SD	7 ± 3
Major anastomotic leak (grade 3), n (%)	
Yes	1 (6.7)
No	13 (86.4)
Conduit necrosis (grade 4), n (%)	1 (6.7)
Organ failure, n (%)	
AKI	1 (6.7)
AF	1 (6.7)
Bronchopneumonia	5 (33.3)
Persistent hyponatremia	1 (6.7)
MODS	1 (6.7)
No organ failure	6 (40)
CPAP ventilation, n (%)	
Yes	7 (46.7)
No	8 (53.3)
Reintubation, n (%)	
Yes	2 (13.3)
No	13 (86.7)
Length of hospital stay (days), median (IQR)	16 (12-24)
Mortality (30 days), n (%)	2 (13.3)

The global QoL of those patients who sustained major postoperative complications was compared with those who had an uneventful postoperative recovery (Table 3). Although it was observed that both groups had considerable differences in the global QoL at one year, there was no statistically significant difference observed (p-value = 0.755). The low sample size may be the reason for this disparity.

**Table 3:** Global QoL comparison at the one-year follow-up between patients with postoperative complications and those who recovered uneventfully Quantitative variables with a normal distribution were expressed as mean± SD and those with a non‐parametric distribution as median (IQR) values. Categorical data were expressed as frequencies (n) and percentages (%). P-value is considered significant when p<0.05. IQR: interquartile range; QoL: quality of life

	Group 1 (without postoperative complications), n=7	Group 2 (with any postoperative complication), n-5	p-value
Global QoL, median (IQR)	83.33 (66.66-100)	66.66 (16.66-100)	0.755

A total of 12 patients who were alive without tumor recurrence in the MIE group responded to the EORTC QLQ-C30 version 3.0 and EORTC QLQ-OES18 at the one-year follow-up. Two out of 15 patients died within 30 days of surgery, one had local tumor recurrence within 12 months of follow-up. A general population control group of 22 healthy bystanders of patients from the Urology ward of the same hospital were surveyed with the EORTC QLQ-C30 version questionnaire and was compared with the study group.

Functional scales comparison - QoL

The patients following MIE for carcinoma esophagus (Group 2) were observed to have a significant difference in physical functioning, role functioning, and social functioning when compared with the general population (Group 1). Cognitive functioning and emotional function were not found to be significantly different (Table 4). No statistical difference was observed in the global health status among the two groups.

**Table 4:** Functional scales comparison of MIE esophagectomy patients with general population, EORTC QLQ-C30 Group 1: General population; Group 2: MIE esophagectomy patients Quantitative variables with a normal distribution were expressed as mean± SD and those with a non‐parametric distribution as median (IQR) values. Categorical data were expressed as frequencies (n) and percentages (%). P-value is considered significant when p<0.05. IQR: interquartile range; MIE: minimally invasive esophagectomy; EORTC QLQ-C30: European Organization for the Research and Treatment of Cancer (EORTC) QoL Questionnaire; QoL: quality of life

	Group 1 (n-22)	Group 2 (n-12)	p-value
Physical functioning, Median (Range)	86.67 (40-100)	16.67 (0-100)	0.001
Do you have any trouble doing strenuous activities?			
Trouble with long walks?			
Trouble taking a short walk outside of the house?			
Do you need to stay in bed or a chair during the day?			
Do you need help with eating?			
Do you need help dressing?			
Do you need help washing yourself or using the toilet?			
Role functioning, Median (Range)	100 (0-100)	16.67 (0-100)	0.001
Were you limited in doing your work?			
Were you limited in pursuing your hobbies/leisure activities?			
Emotional functioning, mean± SD	78.03 ±22.79	88.89 ± 13.91	0.072
Did you feel tense?			
Did you worry?			
Did you feel irritable?			
Did you feel depressed?			
Cognitive functioning, mean± SD	83.33 ± 22.419	81.94 ±20.66	0.430
Have you had difficulty remembering things this past week?			
Have you had difficulty with your daily activities?			
Social functioning, mean± SD	82.57 ± 23.83	65.27 ± 27.94	0.033
Has your physical condition or medical treatment interfered with your family life?			
Has your physical condition or medical treatment interfered with your social activities?			
Global health status, mean± SD	70.454 ± 24.95	76.38 ± 26.312	0.258
How would you rate your overall health?			
How would you rate your overall quality of life?			

Symptom scale comparison - QoL

There was no significant difference found in the general symptoms score comparison of the MIE patients with the general population (Table 5).

**Table 5:** Symptom scale comparison - EORTC QLQ-C30 Group 1: General population; Group 2: MIE Esophagectomy patients Quantitative variables with a normal distribution were expressed as mean± SD and those with a non‐parametric distribution as median (IQR) values. Categorical data were expressed as frequencies (n) and percentages (%). P-value is considered significant when p<0.05. IQR: interquartile range; MIE: minimally invasive esophagectomy; EORTC QLQ-C30: European Organization for the Research and Treatment of Cancer (EORTC) QoL Questionnaire; QoL: quality of life

	Group 1	Group 2	p-value
Fatigue, Median (Range)	22.22 (0-77)	11.11 (0-77)	0.289
Did you need to rest?			
Were you tired?			
Nausea & vomiting, Median (Range)	0(0-100)	0(0-33)	0.792
Have you felt nauseated?			
Have you vomited?			
Pain, Median (Range)	16.67(0-100)	16.67(0-66)	0.193
Have you had pain in the past week?			
Did pain interfere with your daily activities?			
Dyspnea, Median (Range)	0(0-77)	0(0-66)	0.456
Were you short of breath?			
Insomnia, Median (Range)	0(0-66)	0(0-66)	0.748
Have you had trouble sleeping?			
Appetite loss, Median (Range)	0(0-100)	0(0-66)	0.979
Have you lacked appetite?			
Constipation, Median, (Range)	0(0-100)	0(0-33)	0.561
Have you been constipated?			
Diarrhea, Median, (Range)	0(0-100)	0(0-33)	0.922
Have you had diarrhea?			
Financial difficulties, Median (Range)	16.66(0-100)	0(0-66)	0.916
Has your physical condition or medical treatment caused you financial difficulties?			

QLQ-OES18 organ-specific symptom scale

On evaluation of the organ-specific symptom scale, reflux was the one symptom of significant magnitude (score >50), among the patients who survived carcinoma esophagus after undergoing minimally invasive esophagectomy. Dysphagia and dry mouth received low scores, suggestive of minor issues (Table 6).

**Table 6:** EORTC QLQ-OES18 organ-specific symptom scale EORTC QLQ-OES18: European Organization for the Research and Treatment of Cancer QoL Questionnaire - Oesophageal Cancer Module Quantitative variables with a normal distribution were expressed as mean± SD and those with a non‐parametric distribution as median (IQR) values. Categorical data were expressed as frequencies (n) and percentages (%). P-value is considered significant when p<0.05.

	Score, Median	Range
Dysphagia	27.77	0-66.67
Trouble swallowing saliva	0	0-100
Choked when swallowing	0	0-100
Eating	16.67	0-100
Dry mouth	33.33	0-66.67
Trouble with taste	0	0-66.67
Trouble with coughing	0	0-66.67
Trouble talking	0	0-66.67
Reflux	58.33	0-66.67
Pain	0	0-55.5

## Discussion

The current study investigated the clinicopathological profile and the short-term outcomes of patients undergoing MIE, which is the standard of care in the current scenario along with multimodality treatment for esophageal carcinoma. As we observed, unlike Western populations, the younger age group was affected more by carcinoma esophagus in southern India, with a mean age of 50 years. Predominantly male sex was affected, which may affect the economic stability of the family.

In more than 90% of cases, the tumor was confined to the lower esophagus with SCC being the most common histopathological variant. We came across a rare case of primary esophageal lymphoma in the middle 1/3rd in this study. Sixty percent of the study group are current smokers or ex-smokers. While analyzing the postoperative outcomes, nine out of 15 patients were observed to develop major postoperative issues, including pulmonary complications. Major reasons for these complications were prior smoking status, preexisting medical conditions, prolonged periods of nutritional deprivation, post-radiotherapy effects, inadequate prehabilitation, operative complications, and hospital-acquired, and ventilator-associated infections. Deviation from the normal postoperative course increases the duration of ICU stay, additional interventions, treatment expenses, delayed adjuvant therapy as well as delay in regaining normal work-life ease and affects the emotional well-being of patients.

Major anastomotic leak (grade 3) as well as conduit gangrene were observed as serious complications leading to reintervention and mortality. Thoracic anastomotic leak following esophagectomy carries a three times higher death risk than for patients without a leak, and mortality can reach up to 60% [11]. Major determinants for this are conduit vascularity, tension at the anastomosis, level of hemoglobin, intraoperative blood loss, acidosis, prolonged surgery, and lack of surgical expertise [12]. Esophagectomy followed by cervical anastomosis, which was routinely followed in our institution, will not reduce the risk of leak, but avoids the major complications of leak and helps in conservative management of minor leaks. In many studies, cervical anastomosis is considered more risky than thoracic anastomosis as far as leak is concerned [13]. The aftermath of major leaks and conduit non-viability in cervical anastomosis will be turbulent and may develop fistula, sepsis, and death [14].

As the survival of cases managed with multimodality therapy has improved significantly across the globe [6], the newer frontier is about the QoL of the survivors. It can be assumed that short-term complications can significantly affect the QoL of patients. We analyzed the global health QoL among those who recovered from complications with those who had an uneventful postoperative recovery. Though analysis of global health QoL scores of those with postoperative complications and those who had uneventful recovery at one year revealed a higher score for the latter, it was not statistically significant.

A significant difference has been observed in the social functioning, role functioning, and physical functioning between the carcinoma esophagus survivors and the general population in our study. This is contrary to other studies. Svetanoff et al., in their prospective series, state that there was no difference in functional status between the general population and the esophageal resection cohorts [15]. Postoperative reflux remains a significant symptom after esophageal resection, with dysphagia and dry mouth as minor issues. The literature on reflux mitigation during gastric tube construction is ambiguous. The effectiveness of additional procedures such as pyloric drainage, cervical anastomosis, and anti-reflux anastomosis in reducing reflux risk is a matter of debate. Postoperative patients experiencing reflux can undergo evaluation through upper GI endoscopy. Management options include lifestyle and diet modifications, as well as the use of proton pump inhibitors [16]. Our analysis did not reveal any statistically significant distinctions in nausea, vomiting, fatigue, or generalized pain among the patient group we examined. Nevertheless, it's worth noting a potential bias in symptom reporting, as patients who had fully recovered from surgery without any immediate complications tend to report lower symptom scores. It has been reported that the QoL post resection will reach the nadir at two months and slowly improve by the one-year follow-up [17]. Hence further follow-up in subsequent years can help us understand the trend in QoL following MIE in carcinoma esophagus.

Strengths and limitations of the study

This study has some strengths like robust design, strict follow-up of patients, rigorous recording of perioperative parameters and events, validated questionnaires, etc. Limitations of our study include insufficient power to establish the advantage of uneventful postoperative recovery in global health QoL. 

## Conclusions

This study throws light on postoperative complications, which in turn can prolong hospital stays, delay resuming normal work, and affect the global QoL of patients. Physical role and social function were observed to be poor among survived patients of carcinoma esophagus at the one-year follow-up when compared with the normal population. Nutritional prehabilitation, cutting-edge surgical practice including minimally invasive techniques, minimizing deviation from normal postoperative recovery by high-quality ICU care and postoperative rehabilitation are the cornerstones to ensure better QoL.
